# Editorial: Challenges and prospects for conservation genetics at XXI century

**DOI:** 10.3389/fgene.2025.1554590

**Published:** 2025-02-18

**Authors:** Patricia Amavet, Gabriela P. Fernández, Viviana Solís Neffa

**Affiliations:** ^1^ Laboratorio de Genética, Facultad de Humanidades y Ciencias (UNL), CONICET, Santa Fe, Argentina; ^2^ Centro de Bioinvestigaciones (CeBio), Universidad Nacional del Noroeste de la Provincia de Buenos Aires (UNNOBA), CITNOBA (UNNOBA-UNSAdA-CONICET), Pergamino, Buenos Aires, Argentina; ^3^ Instituto de Botánica del Nordeste (IBONE, CONICET-UNNE)-FaCENA (UNNE), Corrientes, Argentina

**Keywords:** population genetics, barcoding, metabarcoding, molecular markers, quantitative genetics, biodiversity conservation

The ongoing biodiversity crisis demands rapid advancement in our knowledge of basic aspects of species, and promoting efficient conservation strategies and policies (Jaureguiberry et al., 2022). In this sense, genetic and genomic analyses have emerged as powerful tools, providing valuable insights into the taxonomic, demographic, biogeographic, ecological, population, and species conservation issues (Hohenlohe et al., 2021). Two decades ago, researchers in genetics faced significant challenges, including limited genetic information on the species under study and the high cost of molecular methods necessary to obtain basic genetic data. However, the scenario has changed considerably. Today, molecular genetic data on diverse life forms are being incorporated into global databases daily. Notably, there has been a remarkable increase in research on these topics within Latin American countries, driven by technological advances and increased access to molecular tools. This trend is clearly reflected in this Research Topic, where contributions from Latin American researchers are prominently represented.

Genomic approaches exploit diversity among DNA sequences to identify organisms. These sequences can be viewed as genetic ‘barcodes’ (Hebert et al., 2003), and are obtained by sequencing a specific gene or genome region that characterizes an organism group. This technique was used by Da Silva et al., to produce the first batch of DNA barcode sequences for the vascular plants of Uruguay. Considering a similar type of analysis, but with greater breadth, metabarcoding enables the identification of species' genetic material from complex samples, such as environmental samples. Mannise et al. utilized metabarcoding to analyze the diet of Neotropical foxes by extracting DNA from feces. This approach exemplifies one of the non-invasive sampling techniques increasingly favored in genetic studies to avoid unnecessary harm to living specimens. The authors’ findings were consistent with previous studies based on morphological identification of consumed items, demonstrating that metabarcoding is as effective as traditional species identification methods. Nonetheless, there are challenges to address, particularly regarding the accuracy of analysis methods, such as the occurrence of false positives or false negatives (Corse et al., 2019). These issues can be prevented by using appropriate universal primers, rigorous negative and PCR controls, and careful analysis of results supported by bibliographic references.

On the other hand, traditional molecular markers, such as nuclear markers (e.g., microsatellites) or mitochondrial markers, continue to be widely used for species identification, as demonstrated in the study by Gonzalez et al. The authors propose the presence of the marsh deer (*Blastocerus dichotomus*) in Uruguay in the recent past, extending the species’ geographic range. These markers have also proven effective in researching the diversity and genetic structure of puma (*Puma concolor*) populations in central and southern Argentina (Mac Allister et al.) allowing the definition of two distinct Management Units (MU) in the south of the species distribution. The combined use of different molecular markers allowed Oklander et al. to explore the evolutionary history of the brown howler monkey (*Alouatta guariba*), and to propose the existence of a single species, and three different MU. The genetic data presented in all these studies are crucial to support management institutions in developing more effective conservation strategies for these species.

In a different line of analysis within the field of quantitative genetics, Bruno et al. present data on candidate genes associated with coat color in the Podolica Italiana Grey Cattle breed. This study integrates medium-density single-nucleotide polymorphism (SNP) array genotype data with bioinformatic analyses of cohort structure using a multi-cohort F_ST_-outlier approach. The work exemplifies the growing relevance of large-scale genetic studies, driven by technological advances in sequencing methods over the past decade. These advances enable the generation of vast genomic datasets, which not only require the training of new generations of bioinformatics researchers but also allow for diverse applications of the results. In this case, the findings have direct implications for animal breeding strategies. Moreover, this analytical approach contributes to a deeper understanding of genetic architecture, with potential applications extending beyond conservation genetics to the fields of animal and human medicine.

Finally, an interesting contribution from Napolitano et al. highlights key challenges for conservation genetics research in Latin America. Based on surveys of individuals involved in conservation, the authors identify the main barriers as a lack of funding and limited access to genetic laboratories. However, as previously mentioned, advancements in sequencing technologies have significantly reduced research costs, while the development of international laboratory networks fosters greater collaboration and resource sharing. These factors provide a promising outlook for overcoming these barriers in the near future, facilitating broader access to genetic research tools and promoting conservation efforts across the region.

This small selection of articles highlights the crucial role of genetic studies in advancing conservation efforts. Genetic analyses contribute to identifying threats to biodiversity, understanding species distribution, detecting hybridization events, and defining conservation units, as exemplified by the work of Van der Valk and Dalén (2024), among others. Collecting genetic data is particularly essential in Latin American countries, where such information remains limited. Expanding genetic knowledge in these regions is essential for developing strategies that optimize the sustainable use and conservation of valuable genetic resources, ultimately supporting biodiversity preservation at both regional and global scales.
